# An optimized permeabilization step for flow cytometry analysis of nuclear proteins in myeloid differentiation of blood cells into neutrophils

**DOI:** 10.1016/j.mex.2019.02.011

**Published:** 2019-02-15

**Authors:** G.M. Viryasova, E.A. Golenkina, V.V. Tatarskii, I.I. Galkin, G.F. Sud’ina, N.V. Soshnikova

**Affiliations:** a Institute of Gene Biology, Russian Academy of Science, Moscow, Russia; b The A.N. Belozersky Institute of Physico-Chemical Biology, Moscow State University, Moscow, Russia

**Keywords:** Flow cytometry, Polymorphonuclear leukocytes, Neutrophils, HL-60, Flow cytometry, Protein detection, Permeabilization

## Abstract

Polymorphonuclear leukocytes (PMNLs) or neutrophils play an important role in the innate immune response. Working with human neutrophils is challenging because these cells are sensitive to changes in the surrounding media and quickly become apoptotic. Meanwhile the experiments with mature neutrophils may be very important for studies of blood function. In this paper we propose an improved technique of flow cytometry nuclear protein analysis with double antibody labeling, which allows direct comparison of protein quantity (overlay histograms) in the primary cells (neutrophils) and progenitor cell lines (line HL-60), to study differentiation process and for other research purposes. We suggest improved technique to analyze and compare nuclear proteins levels in the myeloid differentiation model system (HL-60 cell line) and / or primary human neutrophils. This method was justified with measurement of GFI1 protein expression level, as well-known transcription factor, typical and essential for mature neutrophils.

The key protocol features are as follows:

• Suggested protocol allows simply, direct and correct visual comparison of flow cytometry data in overlay diagrams for myeloid blood cells on various stages of differentiation.
• 70% ethanol permeabilization of neutrophils and HL-60 cells results in lower background fluorescence and better peak resolution than MeOH and Saponin permeabilization.
• Non-specific antibody binding in neutrophils can be efficiently blocked by using 1% BSA and non-immune goat serum.

## Graphical abstract

**Figure:**
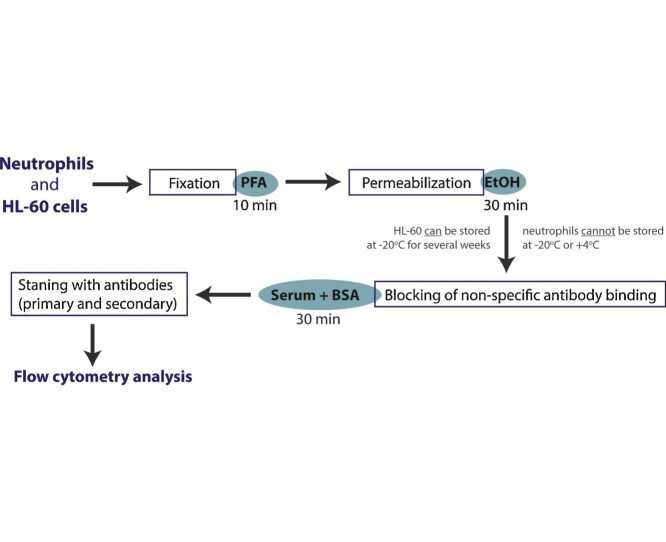

Specifications Table

**Table:** 

Subject area	• Biochemistry, Genetics and Molecular Biology • Immunology and Microbiology
More specific subject area	Protein Detection
Method name	Flow cytometry
Name and reference of original method	P. O. Krutzik and G. P. Nolan. 2003. Intracellular phospho-protein staining techniques for flow cytometry: Monitoring single cell signaling events. Cytometry, vol. 55 A, no. 2, pp. 61–70.
Resource availability	Anti-GFI1 rabbit antibodies, goat anti-rabbit Alexa Fluor 488 antibodies (Thermo Fisher Scientific, Waltham, MA, USA)

## Method details

### Reagents

1 RPMI-1640 medium without sodium bicarbonate (Merck, Darmstadt, Germany)
2 Sodium bicarbonate (Merck, Darmstadt, Germany)
3 HEPES (Merck, Darmstadt, Germany)
4 PBS tablets without calcium and magnesium (Thermo Fisher Scientific, Waltham, MA, USA)
5 Formaldehyde solution, methanol free (Thermo Fisher Scientific, Waltham, MA, USA)
6 Bovine Serum Albumin, BSA (Merck, Darmstadt, Germany)
7 Fetal Bovine Serum, FBS (Merck, Darmstadt, Germany)
8 Non-immune goat serum (Thermo Fisher Scientific, Waltham, MA, USA)
9 Anti-GFI1 (PA5-77985) rabbit antibodies (Thermo Fisher Scientific, Waltham, MA, USA)
10 Goat anti-rabbit Alexa Fluor 488 antibodies (Thermo Fisher Scientific, Waltham, MA, USA)
11 HL60 cells were purchased from collection of ATCC (Manassas, VA, USA)
12 All-trans-retinoic acid (ATRA) (Merck, Darmstadt, Germany)

Additional reagents, used to verify method:

13 Protease inhibitor cocktail cOmplete (Roche Diagnostics, Indianapolis, IN, USA)
14 Phosphatase Inhibitor Cocktail III (Abcam, Milton, United Kingdom)
15 Diisopropylfluorophosphate (DFP) (Merck, Darmstadt, Germany)
16 Z-VAD-FMK (Selleckchem, Houston, TX, USA)
17 Anti-CD66b PE-conjugated antibodies (Becton Dickinson, Franklin Lakes, NJ, USA)

### Equipment

Flow Cytometer, Cytoflex (Beckman Coulter, Brea, CA)

Note: This list does not include any small generic laboratory equipment that is assumed to be available. Chemicals and other components could be used from any reliable company.

## Procedure

### Human neutrophils isolation and HL-60 cell growing

1 Neutrophils were isolated from blood of healthy donors using standard technique with 3% dextran and Ficoll-Paque, which was described previously [1] and confirmed with flow cytometry analysis of CD66b surface marker, specific for mature neutrophils. (Fig. S1, Supplementary)
2 HL-60 cells were grown in RPMI-1640 medium (with HEPES and sodium bicarbonate) with 10% FBS and 2 mM l-glutamine until concentration 1*10^6^ per ml. To model differentiation process HL-60 cells were treated by 2 mM ATRA according to common used protocols [2]. Differentiation of HL60 was confirmed by CD66b flow cytometry analysis. (Fig. S1, Supplementary)
3 Each experimental sample contained 2*10^6^ cells.

Note: Neutrophils could be lost during the sample preparation, so it is better to take a 2–3 times bigger sample.

### Fixation and permeabilization

4 Resuspend 2*10^6^ cells in 5 ml of PBS containing 0.05% BSA, centrifuge (270 *g*, 4 °C, 6 min).
5 Resuspend the pellet in 1 ml PBS with 2–4% formaldehyde (PFA).
6 Incubate at 37 °C for 10 min, then add 5 ml of cold PBS with 0.05% BSA and centrifuge (270 *g*, 4 °C, 6 min).
7 Resuspend the pellet in 1 ml of 70% ice-cold ethanol, place on ice for 30 min. Centrifuge (300 *g*, 4 °C, 6 min).

Note: Add first 200 ul of cold PBS and resuspend the pellet gently. Then add 400 ul of 96% ice-cold ethanol and vortex shortly. HL-60 cells can be stored at −20 °C for up to 3 weeks after 30 min on ice. Neutrophils cannot be stored this way. We recommend proceeding to the next stages of the protocol immediately after the permeabilization of neutrophils.

### Blocking of non-specific binding of antibodies (ab)

8 Resuspend the pellet in 3 ml PBS with 1% BSA and 10% non-immune goat serum.
9 Incubate 30 min at room temperature (RT), centrifuge (300 *g*, 4 °C, 6 min).

Note: For blocking, use the serum of the animal in which secondary Ab were produced.

### Staining with primary ab

10 Resuspend the pellet in 100 ul of PBS with 1% BSA (staining buffer).
11 After 10 min add primary antibodies in required concentration.
12 Incubate 30–40 min at RT.
13 Add 2.5 ml of staining buffer and centrifuge (300 *g*, 4 °C, 6 min).

### Staining with secondary ab

14 Resuspend the pellet in 200 ul staining buffer, containing secondary Ab.
15 Incubate 30 min in the dark at RT, centrifuge (300 *g*, 4 °C, 6 min),
16 Wash with 1 ml PBS, centrifuge (300 *g*, 4 °C, 6 min).
17 Resuspend the pellet in 350 ul PBS and start flow cytometry analysis.

### Method validation

Flow cytometry (FACS) is a well-known method widely used for many tasks, e.g. intracellular and cell surface molecular detection, with such advantages as multiparametric analysis, semi-quantitative data analysis, statistical reliability and relatively easy sample preparation [3]. To evaluate changes in protein expression during differentiation of promyelocytes into leukocytes we created a model system "HL-60 cells - differentiated with trans-retinoic acid (ATRA) HL-60 cell - primary neutrophils" [2]. The HL-60 cells are identified as acute promyelocytic leukemia cells [4]. Presently they were described as promyelocytes or myeloblasts and are well known as model system for studying myeloid cell differentiation [5]. Working with primary human neutrophils is complicated because of many specific properties, high sensitivity to permeabilization agents and impossibility of sample storage. Thus, for this investigation we had to elaborate а universal flow cytometry protocol, which would allow comparison of the nuclear protein expression data obtained in neutrophils and in HL-60 cells. Various activators and transcriptional complexes are responsible for gene expression, which are also changing in the process of differentiation [6]. Changes in the levels of transcription activators may lead to various diseases such as leukemia [7]. To validate suggested protocol improvements, we measured the changes in GFI1 protein expression, which is known to be primarily important in myeloid and lymphoid differentiation. GFI1-deficient mice show a block in myeloid differentiation, an accumulation of myelomonocytic cells, and an expansion of GMPs, which are myeloid progenitors known to cause acute promyelocytic leukemia [8]. GFI1 promotes differentiation in myeloid cells and is crucial for late-stage neutrophil production [9].

Intracellular protein analysis by means of flow cytometry consists of few main steps: fixation, permeabilization, blocking of non-specific binding, staining with antibodies. Here we provide the protocol for two-step staining by primary and secondary antibodies with optimized fixation and permeabilization steps.

Fixation is required for maximum preservation of cell structure. Cells were fixed with 2–4% solution of para-formaldehyde (PFA), without admixture of methanol [10], to avoid the increase of fluorescence intensity [11].

**Fig. 1:**
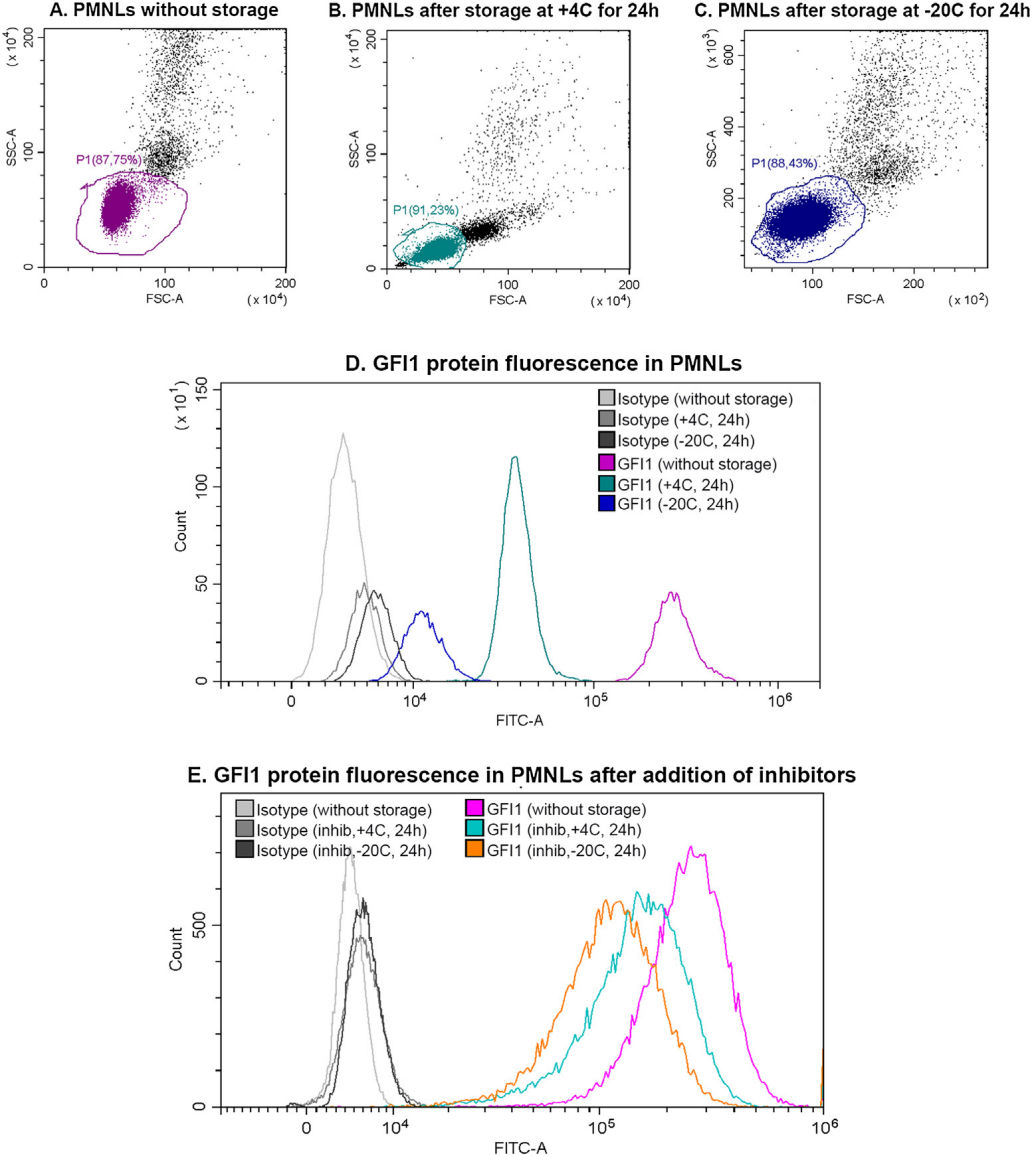
Flow cytometry data for analysis of GFI1 protein in human neutrophils. FSC-SSC plots for neutrophils after permeabilization with 70% EtOH for 30 min on ice (А) or after permeabilization in 70% ethanol and storage of samples for 24 h at + 4 °C (B) and at −20 °C (C). Storage of samples after permeabilization at + 4 °C and −20 °C in case of neutrophils leads to significant changes in scatter (for more that 100 times, plot C) and decrease of fluorescence level after staining with antibodies (D) according to the Protocol. Adding of inhibitors cocktail to buffer solutions leads to smaller decrease of fluorescence level after samples being stored at +4C and -20C (E).

Next step, permeabilization, is used for partial destruction of cellular and nuclear membranes to allow the antibodies to penetrate into the cell. Commonly used permeabilizers are alcohols [3] and detergents. The best choice of reagent depends on its action on the cells and further goals of the experiment. According to assay of Krutzik and Nolan, best separation of intracellular protein peaks in flow cytometry analysis of Jurkat T cells can be reached using alcohol permeabilization (MeOH or EtOH) with PFA fixation. Permeabilization with Saponin or Triton X-100 is possible, but decreases staining intensities [10]. We tested MeOH, EtOH and non-alcohol (saponin) permeabilization in analysis of human leukocytes and their progenitors. We have shown that neutrophils, in contrast to HL-60 cell line, are more sensitive to dehydration. Methanol 70% and 90% (no significant difference between concentrations, data is shown for 90%) as well as 0.1% saponin solution alter the morphological properties of the cells slightly stronger than permeabilization with 70% ethanol (Supplementary file, Fig. S2). The most important fact is that although almost all cells (HL-60 cells as well) can be stored at -20C after ethanol/methanol permeabilization for up to 3–4 weeks [10], neutrophils, however, cannot. Freezing and storage of neutrophils in 70% EtOH or 70–90% MeOH leads to some significant changes in scatter (10^2^ instead of 10^4^ intensity units, see Fig. 1C), isotype fluorescence and decrease of the fluorescence level during the subsequent staining, which makes these results unsuitable for any comparison. Neutrophils cannot be stored after fixation and ethanol treatment at 4C as well, because this also causes changes in scatter and fluorescence level (Fig. 1).

Using EtOH for permeabilization can disrupt the granules in neutrophils, releasing different proteases, phosphatases and myeloperoxidase. To verify if this could be a reason of inability to store PMNLs for the long time, we included different inhibitors, added simultaneously: 2 mM DFP, 50 mkM zVAD, Protease inhibitor cocktail complete (diluted 1:50), Phosphatase Inhibitor Cocktail III (diluted 1:100). These inhibitors were added in buffer solutions before and after permeabilization step. According to our results the presence of inhibitors decreases the loss of fluorescence signal and morphological changes, caused by 24 h storage at +4C or -20C, but still does not allow to achieve the same results as without storage (Fig. 1E).

We recommend to conduct all experiments with neutrophils on the day of the isolation with standard isolation protocol [12].

**Fig. 2:**
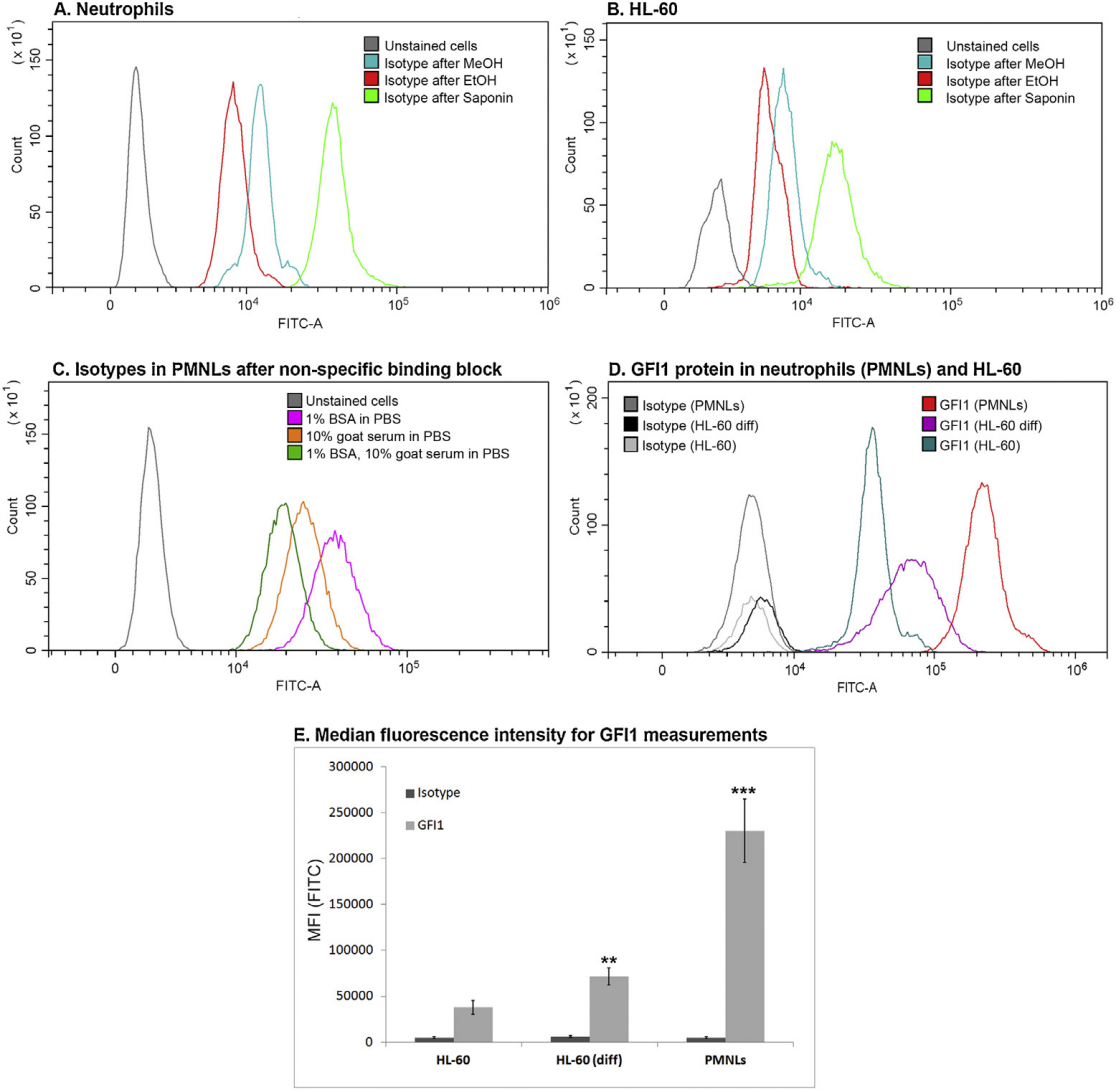
Flow cytometry overlay diagrams demonstrate AlexaFluor488-labelled antibodies distribution in neutrophils after permeabilization with 70% EtOH, 90% MeOH or 0.1% saponin. (A) The efficiency of block of secondary Ab non-specific binding (fluorescence level of isotype control) depending on the permeabilization agents in human leukocytes, primary cells. (B) Isotype controls in case of different permeabilization agents in HL-60 cells. (C) Isotype controls in PMNLs after possible non-specific binding sites were blocked with different reagents: 1% BSA, 10% goat serum or both. (D) The alterations of GFI1 protein content during myeloid differentiation of cell line HL-60 with trans-retinoic acid (ATRA) and in human neutrophils, samples were prepared according to the Protocol. (Е) The median fluorescence intensity data for GFI1 protein. Values indicate the mean ± SD from three independent experiments. ^**^ p < 0.01, ^***^ p < 0.005 compared to the GFI1 content in HL-60. For all pairs Isotype-GFI1b p-value is less than 0.005 (not mentioned on the diagram. Statistical analysis was done with 2-way ANOVA, Holm-Sidak’s multiple comparison test.

Blocking of nonspecific staining is important to make sure that secondary antibodies are not binding to non-specific proteins; otherwise, it can lead to false positive results. To compare the data for primary cells and HL-60 cells we had to choose the conditions providing the same fluorescence level for isotype controls in both cell types. Non-specific binding in HL-60 can be blocked with incubation in 1% bovine serum albumin (BSA) in phosphate buffer solution (PBS) for 30 min before staining. For neutrophils it is better to use combination of 1% BSA in PBS with 10% non-immunized serum of the animal, from which the secondary antibodies were obtained, for 30 min at room temperature (RT), see Fig. 2C. In case of neutrophils permeabilization with MeOH or EtOH leads to more effective blocking of secondary antibodies binding, rather than when using saponin. Fluorescence levels of isotypic controls after different kinds of permeabilization in neutrophils and HL-60 are shown on Fig. 2A, B.

Effects of EtOH could be similar to MeOH and sometimes MeOH treatment with its apparent higher permeability could result in higher fluorescent intensity. We have not detected any significant changes in the case of neutrophils (data not shown). Meanwhile 70% EtOH decreases basic fluorescence, which is then almost similar for both cell types. The main aim of the study was to suggest conditions, which allow to get visually compatible results for cell line HL-60 and primary neutrophils. Therefore, it was important to find experimental conditions, where isotypic controls for all cell types look the same way and have same basic fluorescence intensity. Thus, we used permeabilization with EtOH for further measurements, because of its ability to show the greatest fluorescence ratio of isotypic controls and treated with antibodies cells.

The results of flow cytometric analysis of GFI1 protein with the optimized protocol outlined in this paper are shown on Fig. 2C, in order to determine the validity. Cells were fixed with 2% formaldehyde (PFA), permeabilized with ice-cold ethanol. After possible non-specific binding sites were blocked with 1% BSA and 10% goat serum in PBS, primary rabbit antibodies for GFI1 protein were added, then cells were stained with AlexaFluor488-labeled secondary goat anti-rabbit antibodies.

In this work, we suggested several modifications to flow cytometry protocol for intracellular protein analysis, which help to analyze proteins in human neutrophils and their progenitors. We recommend to permeabilize neutrophils with 70% EtOH and not to store neutrophils at + 4 °C or −20 °C after permeabilization (HL-60 cells can be stored this way).

### Additional information

Suggested sample preparation allows use of flow cytometry for analysis of nuclear proteins in different types of blood cells and eliminates mistakes by direct comparison (overlay histograms) of the results obtained in cell lines and primary culture. Measurements of GFI1 transcription factor, well known as a marker of neutrophil development, was chosen to verify the protocol. GFI1 plays important role in functions of mature neutrophils and its level is increasing during myeloid differentiation [8,13], what was also confirmed by results we obtained with suggested method.

## Author contributions

G.M designed and performed the experiments and wrote the manuscript, E.A. and G.F. supervised the study, I.I. assisted with some measurements, V.V and N.V. corrected the manuscript and provided antibodies.

## Competing interests

The authors declare no conflict of interests.
