# Supplementary material for: An optimized permeabilization step for flow cytometry analysis of nuclear proteins in myeloid differentiation of blood cells into neutrophils
Source: MethodsX. 2019 Feb 15;6:360–7. doi: 10.1016/j.mex.2019.02.011 (PMC6396090; doi:10.1016/j.mex.2019.02.011)
Supplement: Supplementary file 1 [file mmc1.docx]

**Supplementary file**

for the paper MEX-D-18-00458 “An optimized permeabilization step for flow cytometry analysis of nuclear proteins in myeloid differentiation of blood cells into neutrophils”


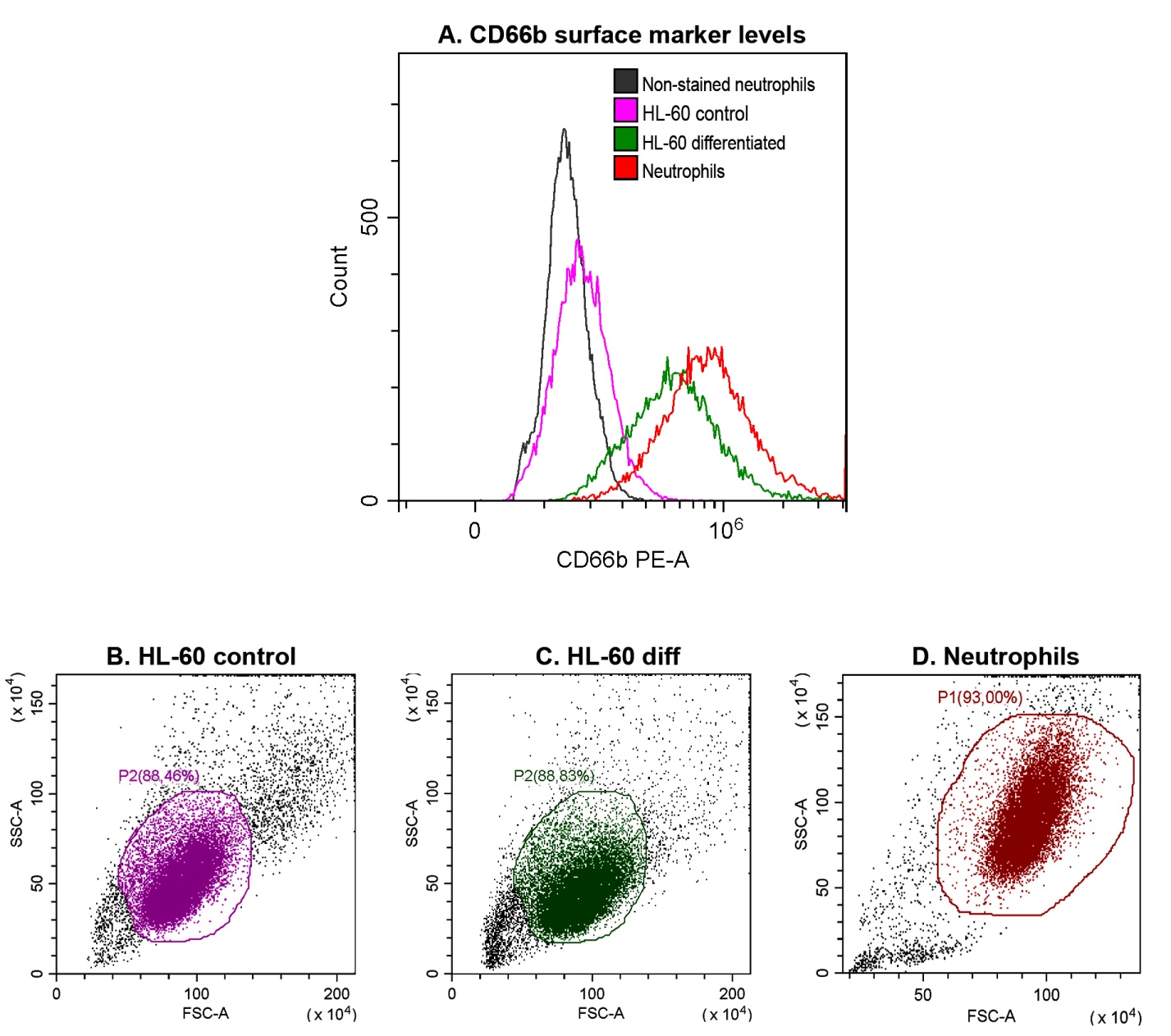


Fig. S1. Differentiation of HL-60 cells compared to freshly isolated human neutrophils. CD66b neutrophils-specific surface marker was measured by flow cytometry (A), also FSC-SSC diagrams are shown (B, C, D).


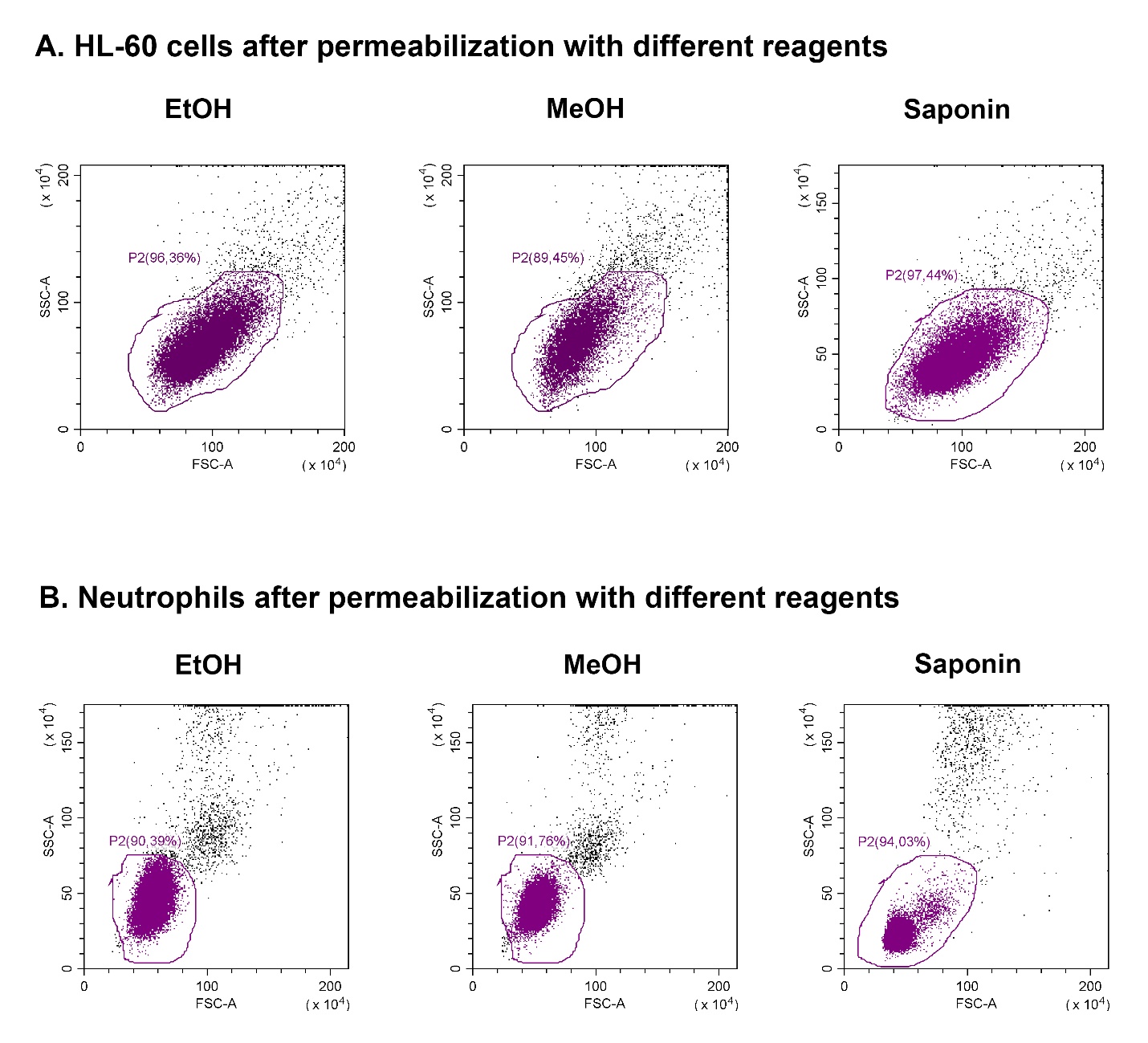


Fig. S2. FSC-SSC flow cytometry diagrams for HL-60 cells (A) and neutrophils (B) after permeabilization with different reagents: 70% EtOH, 90% MeOH and 0.1% saponin.
